# A Rare Case of Acute Liver Failure Secondary to Diffuse Hepatic Infiltration of Small Cell Neuroendocrine Carcinoma

**DOI:** 10.3389/fonc.2021.616337

**Published:** 2021-03-04

**Authors:** Ping Yan, Yu Liu, Qing Wang, Xia Chen

**Affiliations:** ^1^ Department of Gastroenterology, Affiliated Hospital of Southwest Medical University, Luzhou, China; ^2^ Department of Hepatobiliary Surgery, People’s Hospital of Leshan, Leshan, China

**Keywords:** acute liver failure, biopsy, pulmonary neuroendocrine tumor, small cell lung cancer, case report

## Abstract

**Background:**

Malignant liver infiltration is an uncommon cause of acute liver failure (ALF) and has rarely been reported.

**Case Presentation:**

We present a patient with progressive jaundice and dissociation of bilirubin and aminotransferases, who had no history of relevant liver diseases or tumor except the use of Chinese traditional drugs for a cold. An abdominal computed tomography (CT) scan showed ascites without hepatic focal lesions. Laboratory studies revealed no evidence of hepatitis or underlying autoimmune disorders. Following 8 days of conservative management ALF rapidly worsened. Contrast-enhanced CT revealed diffuse regenerative nodules in the liver. The patient underwent liver biopsy, which demonstrated that the liver was infiltrated by pulmonary neuroendocrine tumor classified as small cell lung cancer. The patient died 13 days after diagnosis.

**Discussion and Conclusions:**

This case represents a rare cause of ALF induced by pulmonary neuroendocrine tumor of small cell type and illustrates the importance of prompt biopsy in an unknown cause of ALF.

## Background

Acute liver failure (ALF) is defined as acute liver dysfunction manifesting as encephalopathy and coagulopathy [INR (international normalized ratio)] ≥ 1.5) of less than 26 weeks duration, without preexisting liver disease (1). Of the known etiologies of ALF in adults, drug toxicity (50%), viral hepatitis (9%), and autoimmune hepatitis (7%) are most common (2, 3). Although the liver is a common target for metastasis, a significant number of patients are asymptomatic with mildly abnormal liver function tests. There are very few reports of ALF resulting from malignancy (0.44–1.4%) (3, 4). Hematologic malignancies are the leading cause of ALF, especially non-Hodgkin lymphoma (3, 5).

The diagnosis of widespread infiltration of the liver can be challenging, as imaging and clinical presentations often do not reveal this type of hidden infiltration pattern (6). However, most cases have a poor prognosis with liver failure occurring within several days (2). Early liver biopsy in unexplained cases must be carried out, as the findings can provide information on appropriate treatment.

We report a case of ALF associated with malignant infiltration of small cell neuroendocrine carcinoma without a history of primary malignant tumors. We evaluated the clinical and laboratory data, treatment and prognosis.

## Case Presentation

### Chief Complaints

A 69-year-old man was admitted to a community hospital with abdominal pain, bloating, and burning under the xiphoid of 1 week duration. His symptoms worsened following the discovery of liver dysfunction 5 days later and he was then transferred to our hospital. He complained of intolerable abdominal distension and decreased appetite on admission.

### History of Past Illness

His medical history included hypertension treated with antihypertensive drugs for 14 years. He had also undergone laparoscopic appendectomy 20 years previously. There was no history of excessive alcohol consumption or hepatitis. It was noted that he took several types of Chinese traditional drugs (Qinghao Biejia Decoction) for a cold.

### Physical Examination

The patient had jaundice. The lungs were clear, and his heart rate was normal. His upper abdomen was tender, but there was no organomegaly. The bowel sound was weakened with negative shifting dullness.

### Laboratory Examinations

Blood tests showed leukocytosis of 13.73 × 10^9^/L (normal range: 3.5–9.5×10^9^/L) and neutrophilia of 12.07 × 10^9^/L (1.8–6.3×10^9^/L) with a normal red cell count and platelet count. Liver function tests demonstrated an anomalous pattern, with elevated aminotransferases/aspartate aminotransferase (ALT/AST) 285.3 U/L (15–40 U/L), ALT 481.8 U/L (9–50 U/L), alkaline phosphatase 471.4 U/L (45–125 U/L), γ-glutamyl transferase 1,424.1 U/L (10–60 U/L), total bilirubin (TB) 90.5 µmol/L (0–23 µmol/L), direct bilirubin (DB) 75 µmol/L (0–7 µmol/L), prolonged prothrombin time of 16.1 s, and INR of 1.19 (0.8–1.2). IgE level was 123.8 IU/ml, and the levels of serum amylase, autoantibody profile, and viral serology (HAV, HBV, HDV, HEV, EBV, CMV, HSV) were normal. Gastrointestinal marker carbohydrate antigen-199 was 90.92 U/mL (0–37 U/ml) and FER was 802.5 ng/ml (25–280 ng/ml).

### Imaging Examinations

Abdominal X-ray showed incomplete small bowel obstruction. A computed tomography (CT) scan of the chest and abdomen revealed exudation of the bilateral lungs, and small pleural effusion with enlarged lymph nodes in the mediastinum (**Figure 2A**), peritonitis, and massive pelvic fluid (**Figure 2B**). The biliary ducts were not dilated.

## Final Diagnosis and Treatment

**Figure 1:**
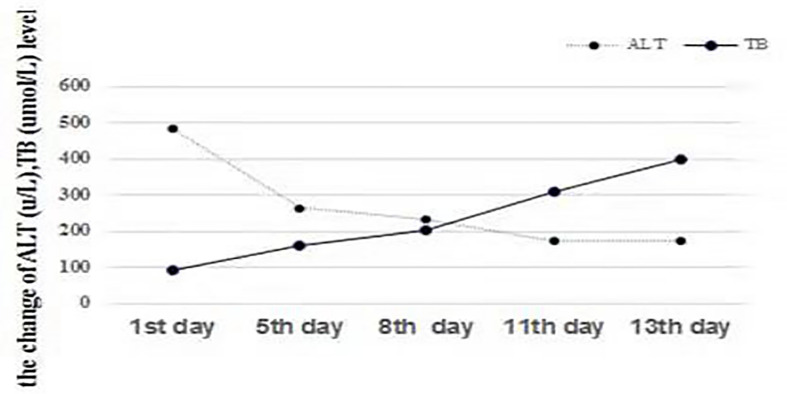
Changes in ALT (U/L) and TB (µmol/L). The level of TB consistently increased with a decrease in ALT, indicating the dissociation of bilirubin and aminotransferases, which represents liver function deterioration.

**Figure 2:**
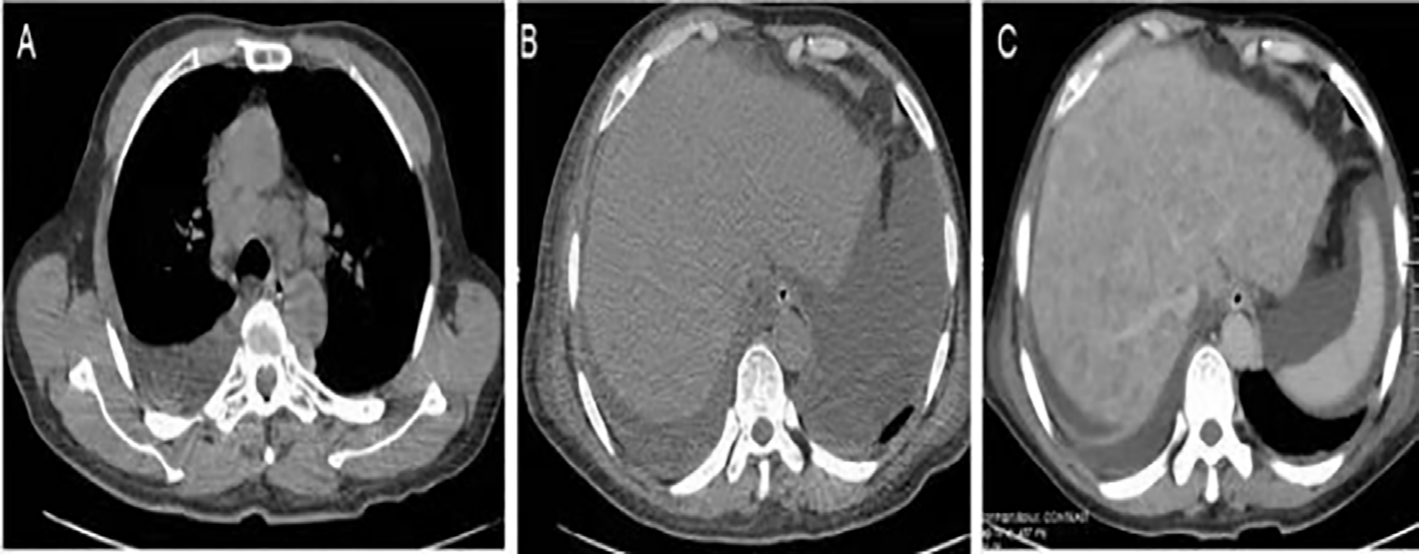
Imaging findings. **(A)** Chest CT revealed enlarged lymph nodes in the mediastinum. **(B)** Abdominal CT showed ascites with no hepatic focal lesions. **(C)** Contrast-enhanced CT of the abdomen revealed diffuse regenerative nodules, cholecystitis, peritonitis, and ascites. No splenomegaly, biliary obstruction, or pulmonary nodules were observed.

**Figure 3:**
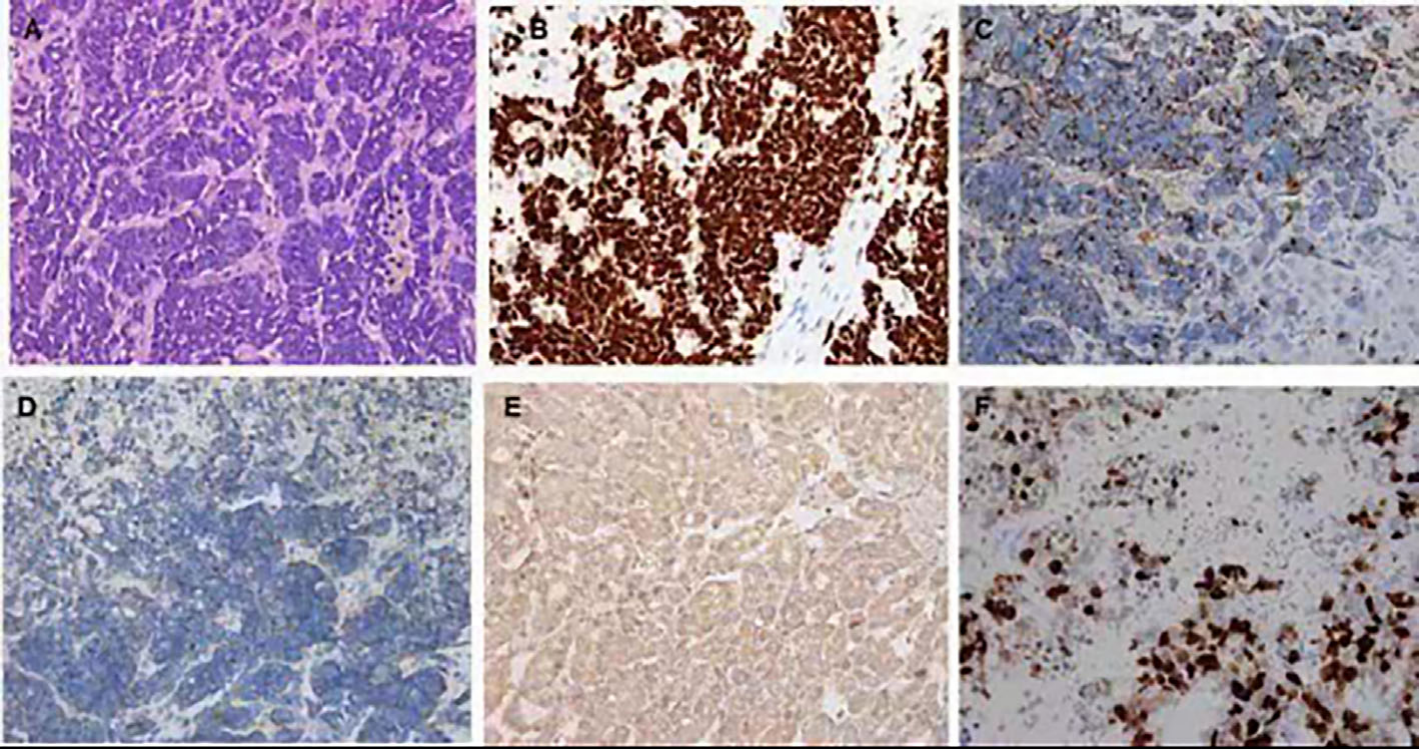
Hematoxylin and eosin and immunohistochemistry staining of the liver biopsy specimen. **(A)** In the center of the field, diffuse infiltration by a poorly differentiated neuroendocrine carcinoma and arranged singly, in small clusters (hematoxylin and eosin ×400). Positive immunohistochemistry staining for TIF **(B)**, CgA **(C)**, Syn **(D)**, CD56 **(E)**, Ki-67 **(F)**.

An extensive workup including abdominal CT, viral serology, and autoimmune studies failed to show an etiology in this patient. A presumed diagnosis of liver dysfunction due to drug-induced hepatitis was made as the patient had taken Chinese traditional drugs before admission, which had nothing to do with ALF or carcinoma infiltration according to current literatures. The patient was treated with liver protective agents and diuretics. On the 8th day, the patient reported aggravated abdominal distension and jaundice. Liver function tests had also deteriorated with increased serum bilirubin level and reduced serum aminotransferase level. Laboratory indices were as follows: TB 201 µmol/L, DB 163.5 µmol/L, ALT 232.7 U/L, AST 213 U/L, and ALB 33.3 g/L, respectively (**Figure 1**). However, subsequent abdominal CT (**Figure 2C**) revealed diffuse regenerative nodules, cholecystitis, peritonitis, and ascites. No splenomegaly, biliary obstruction, or pulmonary nodules were observed (**Figure 2C**). Liver biopsy in multiple sections was performed after patient permission. On the 11th day, the patient developed sleep disorder, abnormal behavior with a decrease in calculation ability, and no fever, digestive tract hemorrhage, or other significant clinical findings were noted. Laboratory indices were TB 307.2 µmol/L, DB 256.1 µmol/L, ALT 173.4 U/L, AST 231.2 U/L, and ALB 32.4 g/L (**Figure 1**), while psychometric and serum ammonia 56 umol/L was normal. The patient then received N-methyl-D-aspartate (NMDA) ornithine, arginine, branched chain amino acid injection, and vinegar for clyster. The use of artificial liver treatment was also considered for this patient. But the liver biopsy results revealed high-grade neuroendocrine carcinoma originating from the lung, classified as small cell type, accompanied with cell necrosis and eosinophilic infiltration in the adjacent liver parenchyma. Immunohistochemical staining was positive for CgA, Syn, Ki-67, TTF-1, CD56, P53, CK19 showed dense positive brown nuclear immunoreactions and the distribution of which positive cells was not uniform in one slide or a few of cancer cells (>10%), while CK7, CK20, P40, GPC-3, HepPar1 were negative without cytoplasmic immunoreactions (**Figure 3**).

## Outcome and Follow-Up

The patient’s clinical course continued to worsen. He refused further examination and treatment, and left the hospital on the 13th day. The patient died 10 days after hospital discharge.

## Discussion

We report a rare case of ALF due to infiltration by a pulmonary neuroendocrine tumor (PNET). To our knowledge, ALF secondary to malignant infiltration of the liver is unusual (3). The diagnosis of ALF secondary to malignancy can be difficult as standard laboratory values are not helpful in identifying the presence of malignancy. As in our patient, only a common clinical presentation was observed (1). Chest CT did not show a lung nodule and only revealed enlarged lymph nodes in the mediastinum. Abdominal CT revealed a nodular liver that has previously been described as “pseudocirrhosis” (6) (**Figure 2B**). The final diagnosis required liver biopsy, in the absence of previous tumors. Our patient underwent a confirmatory biopsy, highlighting the importance of early tissue sampling. It is also important, in terms of both early diagnosis and prompt initiation of treatment, to differentiate between related prodromal symptoms associated with the underlying disease and those associated with ALF. The patient had malaise and nausea for 2 weeks before ALF was diagnosed, and these symptoms are often neglected by patients as they were in our case.

The underlying etiology of ALF with malignant infiltration includes mainly non-Hodgkin’s lymphoma and Hodgkin’s disease (3, 4). Scattered reports show that metastatic carcinoma from lungs (3, 7) and breast represents a rare cause. Our case is distinct from previous reports of ALF due to malignant infiltration of the liver from PNET. PNETs are divided into four major types: small cell lung cancer (SCLC), large cell neuroendocrine carcinoma, atypical carcinoid, and typical carcinoid. The liver biopsy in our case confirmed that the PNET responsible for ALF was the SCLC type, which has never been reported before. PNET has been described to metastasize to other organs and cause the secretion of various hormones, but it is unusual to manifest as ALF. Early liver biopsy with prompt immunostaining is necessary to determinate the diagnosis to ensure appropriate treatment. Lung NETs of the SCLC subtype at stage IV have a poor prognosis and are associated with shorter survival time (8). The Ki-67 level is associated with the degree of differentiation, prognosis, and survival rate (9). Our patient showed 90% Ki-67 staining. Surgery remains the only choice for cure (10), although most patients are diagnosed with metastatic disease, and curative surgery is usually not possible. Long-term systemic treatment with somatostatin analogs and peptide receptor radionuclide therapy alone or in combination can be given to patients with advanced disease who are unsuitable for surgery. Although thorough evaluation of the patient, ideal timing of treatment initiation, and the administration of various regimens are difficult (11), successful diagnosis and prompt treatment have been shown to increase survival and, to a certain extent, be beneficial for symptomatic relief.

## Conclusion

Most patients with ALF due to neoplastic invasion have a dismal prognosis. The mortality rate of diffuse hepatic tumor infiltration varies from 3 days to 6 months after presentation (12). It should be noted that only accurate histological diagnosis following liver biopsy and early initiation of specific therapy in such patients will provide the best chance of recovery.

## Author Contributions

PY reviewed the literature and contributed to manuscript drafting, analyzed and interpreted the imaging findings. XC contributed to manuscript drafting and was responsible for revision of the manuscript for important intellectual content. YL and QW contributed to the analysis, interpretation, critical revision, and final approval of the manuscript. All authors contributed to the article and approved the submitted version. These authors (PY and YL) contributed equally to this work.

## Conflict of Interest

The authors declare that the research was conducted in the absence of any commercial or financial relationships that could be construed as a potential conflict of interest.

## Abbreviations

ALF, acute liver failure; CT, computed tomography; SCLC, small cell lung cancer; PNET, pulmonary neuroendocrine tumor.
